# Inhibition of hyperprogressive cancer disease induced by immune-checkpoint blockade upon co-treatment with meta-tyrosine and p38 pathway inhibitor

**DOI:** 10.1186/s12885-022-09941-2

**Published:** 2022-08-03

**Authors:** Daniela R. Montagna, Alejandra Duarte, Paula Chiarella, Bárbara Rearte, Oscar D. Bustuoabad, Mónica Vermeulen, Raúl A. Ruggiero

**Affiliations:** 1 Laboratory of Experimental Oncology, Instituto de Medicina Experimental (IMEX-CONICET), Academia Nacional de Medicina de Buenos Aires, Buenos Aires, Argentina; 2 Laboratory of Experimental Immunology, IMEX-CONICET, Academia Nacional de Medicina de Buenos Aires, Buenos Aires, Argentina; 3 Laboratory of Physiology of Inflammatory Processes, IMEX-CONICET, Academia Nacional de Medicina de Buenos Aires, Buenos Aires, Argentina; 4 Laboratory of Antigen Presenting Cells and Inflammatory Response, IMEX-CONICET, Academia Nacional de Medicina de Buenos Aires, Buenos Aires, Argentina

**Keywords:** Hyperprogressive cancer disease, Immune checkpoints inhibitors, Meta-tyrosine, Metastases, Murine tumors

## Abstract

**Background:**

Although immune-checkpoint inhibitors (ICI) are overall promissory for cancer treatment, they entail, in some cases, an undesired side-effect called hyperprogressive-cancer disease (HPD) associated with acceleration of tumor growth and shortened survival.

**Methods:**

To understand the mechanisms of HPD we assayed the ICI therapy on two murine tumors widely different regarding immunogenicity and, subsequently, on models of local recurrences and metastases of these tumors. To potentiate the immune response (IR), we combined ICI with meta-tyrosine—that counteracts immune-suppressive signals—and a selective inhibitor of p38 pathway that proved to counteract the phenomenon of tumor-immunostimulation.

**Results:**

ICI were therapeutically effective against both tumor models (proportionally to their immunogenicity) but only when they faced incipient tumors. In contrast, ICI produced acceleration of large and residual tumors. The combined treatment strongly inhibited the growth of large tumors and it managed to cure 80% of mice with local recurrences and 60% of mice bearing residual metastases.

**Conclusions:**

Tumor enhancement was paradoxically correlated to a weak increase of the antitumor IR suggesting that a weak IR – different from a strong tumor-inhibitory one—may produce stimulation of tumor growth, mimicking the HPD observed in some clinical settings.

**Supplementary Information:**

The online version contains supplementary material available at 10.1186/s12885-022-09941-2.

## Background

In the last 20 years, different novel immunological strategies were developed to enhance the basal anti-tumor immune response evoked by growing tumors as well as to counteract the tumor-associated negative immune-regulatory mechanisms that could down-regulate such response [1–4].

Among these strategies, blockade of immune-checkpoints has been considered the most promising one and a potential revolution for the treatment of clinical cancer [5].

A large body of evidence demonstrates that experimental growing murine tumors may be inhibited or even eradicated upon treatment with inhibitors of the cytotoxic T-lymphocyte-associated protein (CTLA)-4 and the programmed cell death-1 (PD-1)/PD ligand-1(PD-L1) pathway.

These treatments have also increased the overall survival and progression-free survival of humans affected by some cancers such as melanoma and non-small cell lung, colo-rectal, and renal cell carcinoma [6–8]. However, some treated patients show no improvement and a variable fraction of them exhibits a condition called ‘hyperprogressive cancer disease’ (HPD) associated with sharp acceleration of tumor growth, worse prognosis, and shortened survival times [9–11]. In addition, some initial responders eventually develop resistance to therapy, and others may be afflicted by immune-related adverse events [12].

Two main arguments could be invoked to explain the disparity between the resounding successes achieved in experimental models and the more modest results observed in clinical settings.

First, while experimental models are usually strongly immunogenic chemically-induced tumors, most human tumors may exhibit significantly lower immunogenicity. In support of this contention, murine tumors of spontaneous origin – which have been considered the best models for common human cancers – usually exhibit weak or undetectable immunogenicity [13–15].

The second argument is related to the fact that immune-checkpoint inhibitors (ICI) were truly effective in restraining experimental murine tumors, although only when they faced incipient tumors. Afterwards, as the tumor becomes larger, and resembles the size that could usually be detectable at first clinical inspection, null, or even stimulatory effects on tumor growth have been observed [16].

It could be argued that immunologic strategies are second-line therapies after surgery, radiotherapy, or chemotherapy have reduced tumor burden. In such cases, residual tumors associated with local recurrences or metastases may be made up of a small number of tumor cells that would mimic an incipient tumor against which ICI proved to be effective. However, despite their similarity concerning the number of cells, both incipient and residual tumors might behave utterly different in terms of their sensitivity to immunologic treatments. In effect, cells from incipient tumors are starting to grow in an otherwise healthy host while cells from a residual tumor are placed in a microenvironment that could have been drastically altered by the previous presence of a tumor.

If the arguments above mentioned were valid, the use of appropriate tumor models that closely resemble the real clinical situation might be helpful to understand – in controlled conditions—both the scope and the limitations of these anti-tumor immunologic treatments [17].

In this work we have evaluated firstly the efficacy of ICI (as well as classical antitumor vaccines) in function of target immunogenicity. For this purpose, we used two murine tumors, a strongly immunogenic methylcholanthrene-induced fibrosarcoma and a weakly immunogenic and highly metastatic mammary carcinoma of spontaneous origin. In a second place, we have analyzed whether the inhibitory effect of ICI on incipient tumors is a good predictor of the outcome of this therapy on local recurrences and metastases after surgical tumor removal. Finally, we investigated the growth-accelerating effect of ICI on large experimental tumors with the hope to understand the up to date elusive underlying mechanisms of the HPD phenomenon observed in clinical settings. To account for this aim, we have considered the possibility that the antitumor immune response may be not linear – as orthodoxy predicts—but biphasic – as the immunostimulatory theory of cancer states—with strong immune responses producing inhibition while weak ones inducing stimulating effects on tumor growth (Suppl. Figure 1) [16, 18, 19, 20]. We suggest that ICI treatment on large tumors produces a weak tumor-stimulating antitumor immune response. Herein, in order to strengthen that weak response and turn it into an inhibitory one, we propose to combine ICI-therapy with two different but complementary strategies, the use of meta-tyrosine and that of a specific inhibitor of p38. Meta-tyrosine is an unnatural isomer of tyrosine that has recently been demonstrated to be capable of rescuing the organism from states of immunosuppression by mechanisms different from the currently known ICI [18]. In consequence, it might exert a boosting and adjuvant like-effect on the antitumor immune response. On the other hand, a specific inhibitor of p38 pathway might counteract the tumor- accelerating effect induced by ICI on the basis that, in a former paper [16], we have suggested that the enhancing effect induced by a weak antitumor immune response was associated with the activation of TLR4 and p38 signaling pathways in macrophages recruited at the tumor place.

## Materials and methods

### Animals

Female and male BALB/c mice were bred in the Academia Nacional de Medicina de Buenos Aires facilities. They were used at 2–3 months of age and 20–25 g of weight. Nude BALB/c mice and NOD Scid Gamma (NSG) mice were purchased from Comisión Nacional de Energía Atómica and Instituto de Biología y Medicina Experimental, Argentina, respectively. Care of mice, an early experimental endpoint and all methods were according to NIH Guide for the Care and Use of Laboratory Animals. All experimental protocols were approved by the Committee for the Care and Use of Laboratory Animals (CICUAL) of Instituto de Medicina Experimental (IMEX-CONICET, protocol N°005/15). All methods were performed in accordance with ARRIVE guidelines. Randomization was carried out by Rand() in software Excel and in vivo experiments were blinded. Animals were euthanized by cervical dislocation after anesthesia with ketamine (100 mg/kg) and xylazine (15 mg/kg) i.p.

### Murine tumors

LMM3: highly metastatic mammary carcinoma, kindly provided by Dr. L. Colombo (Instituto Angel Roffo, Buenos Aires, Argentina).

MC-C: strongly immunogenic fibrosarcoma induced by the chemical 3-methylcholanthrene.

More details of tumor models and surgical procedures have been reported previously [1, 21–23]. Tumor dose 50 (TD50): number of tumor cells able to grow in 50% of mice. Tumor volume was calculated as 0.4ab^2^, where *a* and *b* are the larger and smaller diameters, respectively [21, 22]. We defined incipient, mid-sized, and large-sized tumors, those whose volumes were ≤ 10 mm^3^, 100–400 mm^3^, and > 500 mm^3^, respectively. The medium was RPMI 1640 (Gibco) supplemented as described [1]. Tumor lysates, bone marrow-derived dendritic cells isolation, splenocytes isolation, and histological analyses were performed as previously reported [1, 24, 25]. Tumor growth rate (TGR) was expressed as the increase of tumor volume per unit time = tumor volume at day B—tumor volume at day A / B – A [9–11].

### Reagents

HGMB1 and HSP60 were quantified using ELISA kits from Pepro-Tech, following the manufacturer’s recommendations. TNF-α, IL-12p70, IL-10 and TGF-ß were quantified using ELISA kits from R&D Systems.

### Tumor vaccination strategies

Pre-treatment with X-lethally irradiated (LI) tumor cells and pre-treatment with dendritic cells (DC) incubated with tumor lysate were carried out as reported [1, 25–27].

### Drugs, ICI and radiotherapy

DL-m-tyrosine (Sigma-Aldrich) and p38 inhibitor SB202190 (Santa Cruz Biotechnology) were used as described [18, 28]. JSI-124 (Indofine Chemical Company, Hillsborough, NJ), an inhibitor of STAT3; JQ1 (Sigma-Aldrich), an inhibitor of PD-L1; blocking anti-mouse PD-L1, clone 10F.9G2 and anti-mouse CTLA-4 (CD152), clone 9H10 (BioXCell), were used as reported [29–31]. Treatments with vincristine and radiotherapy (2000 grades in the tumor area; Philips 250/15 radiotherapy device at 220 kV, 14 mA) were used as described [1].

### Flow cytometry

Dendritic cells were incubated with different combinations of the mAbs: anti-CD11c, anti-IAd (MHC class II), anti-CD86 and anti-CD80, following the manufacturer’s recommendations. Splenocytes, and/or tumor cells were incubated with antibodies: anti CD3, CD4, CD8, CD11b, PD-1, PD-L1—clone MIH5 -, phosphorylated-STAT3 (pSTAT3) from Ap-Biotech, Argentina, and 5,6 Carboxi Fluorescein diacetate Succinimidyl Ester (CFSE) from Molecular Probes; Eugene, OR, USA. Fluorescence of individual cells was measured in a flow cytometer (Becton Dickinson) and was analyzed by Flowing (Software version 2.5.1, Turku Centre for Biotechnology. University of Turku, Finland). More details were given elsewhere [1].

### Proliferation assays

Lymphocyte proliferation was evaluated by CFSE staining (Molecular Probes). Briefly, 1 × 10^7^cells/ml were suspended in 0.3% BSA/PBS. Then, 1 μl of CFSE was added for each ml (0.5 μM) (Invitrogen) and cells were incubated for 15 min at 37 °C. Cells were washed three times with complete RPMI and incubated for 5 min at 37 °C between washes. Afterward, 1 × 10^5^ lymphocytes were cultured in 96-well flat-bottom plates for 24, 48, or 72 h in the presence or absence of 3 × 10^3^ DC pre-treated with tumor lysates and/or m-Tyr. Then, 30,000 events were collected and CFSE low expression (proliferating lymphocytes, FL-1) was analyzed by flow cytometry as described above.

### Western blotting

Western blotting was carried out with standard techniques as described and analyzed by ImageQuant software. The following antibodies were used: anti–p-STAT3, anti-STAT3 (Santa Cruz Biotechnology, Paso Robles, CA), anti-p38 (Santa Cruz Biotechnology) and anti–β-actin (Cell Signaling Technology, Danvers, MA). Levels of each band were normalized with β actin densitometry units as reported [16].

### Statistical analysis

Student's t-test, ANOVA, Mann Whitney U test and Kaplan–Meier estimator for survival curves were used. Values were expressed as mean ± standard error (SE). Differences were considered to be significant whenever the *P*-value was 0.05 or smaller.

## Results

### Different properties of tumor models and preventive immunologic strategies

**Fig. 1:**
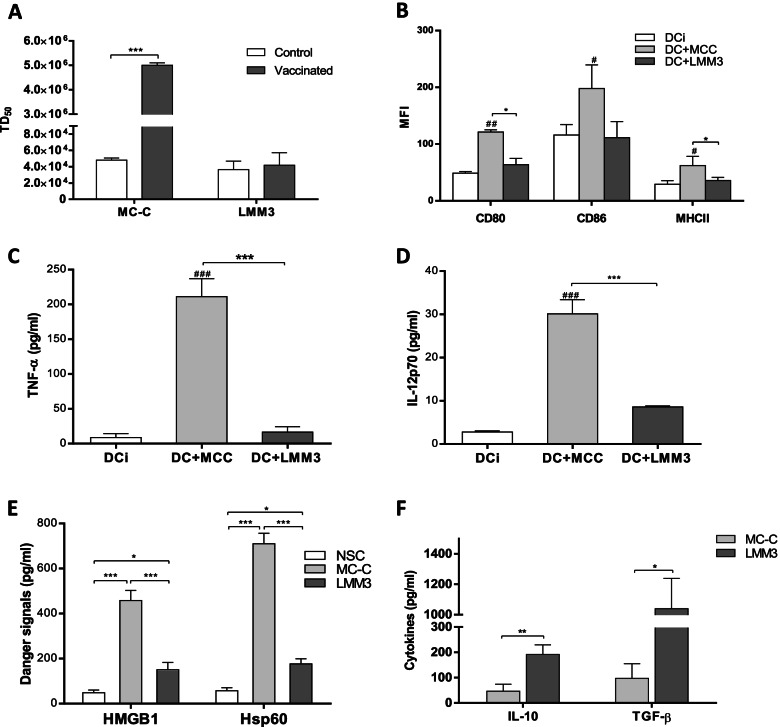
Immunologic properties of MC-C and LMM3 tumors.** (A):** Vaccinating capacity of dendritic cells (DC) stimulated in vitro with MC-C or LMM3 lysates. 2 × 10^5^ DC were inoculated in the footpad of mice 14 and 7 days before the s.c. challenge with different doses of tumor cells. The vaccinating capacity was measured as an increase of tumor dose 50 (TD_50_) of tumors in vaccinated mice compared to control mice. Data represent the mean ± SEM of two independent experiments. In each experiment, 20–25 mice per group were utilized. Similar results were obtained when lethally-irradiated tumor cells were used as a vaccination strategy. **(B):** Expression of cell-surface receptors CD80, CD86, and MHCII in DC stimulated with tumor lysates, evaluated by flow cytometry. Controls were unstimulated DC (DCi). DC stimulated with lysate of normal spleen cells (NSC) displayed similar results to that obtained with DCi, and, for simplicity, they were omitted. MFI = Mean fluorescence intensity. Data represent the mean ± SEM of two independent experiments. **(C, D):** Concentration of TNF-α **(C)** and IL-12p70 **(D)** (pg/ml) in supernatants of DC stimulated with MC-C or LMM3 tumors lysates. Data represent the mean ± SEM of three independent experiments. **(E)** Concentration of danger signals HGMB1 and Hsp 60 (pg/ml) in MC-C and LMM3 tumors lysates and in a NSC lysate. Each value represents the mean ± SEM of three assays. **(F)** Concentration of cytokines IL-10 and TGF-ß in MC-C and LMM3 tumors lysates. Levels of IL-10 and TGF-ß in NSC were undetectable. Each value represents the mean ± SEM of three assays. ELISA assays evaluated cytokines and danger signals levels. Statistical comparison between: experimental groups vs. DCi: # *p* < 0.05; ## *p* < 0.01; ### *p* < 0.001. Statistical comparison among the experimental groups:* *p* < 0.05; ** *p* < 0.01; *** *p* < 0.001.

MC-C tumor proved to be strongly immunogenic as far as pre-treatment of mice with lethally-irradiated MC-C tumor cells or with DC stimulated with MC-C tumor lysate strongly prevented the growth of live MC-C tumor cells implanted thereafter (Fig. 1A). This effect was tumor-specific and T-cell dependent (not shown), indicating specific and robust tumor antigens.

**Fig. 2:**
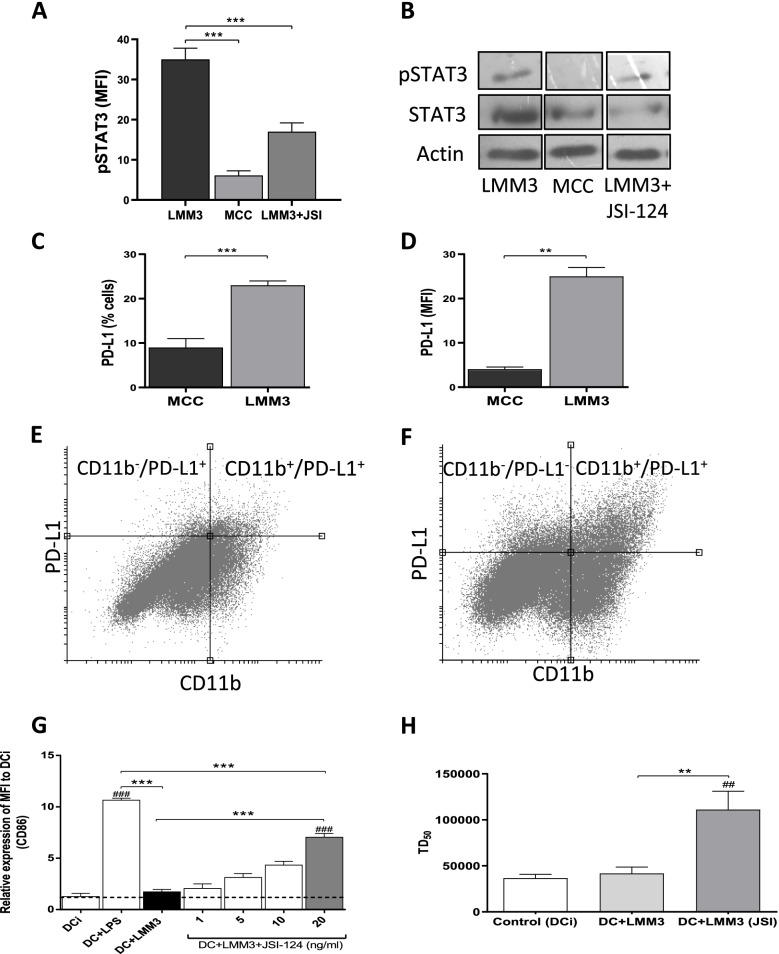
Expression of activated STAT3 (pSTAT3) and PD-L1 in MC-C and LMM3 tumors. pSTAT3 was evaluated by flow cytometry **(A)** and Western Blotting **(B)** in LMM3, MC-C, and LMM3 tumor cells that have been treated with 20 ng/ml of JSI-124 for 24 h. Controls with actin and total STAT3 were added. Results are representative of three similar experiments. **(C, D)** Percentage of cells PD-L1^+^ **(C)** and mean fluorescence intensity (MFI) **(D)** in MC-C and LMM3 tumors. Each value represents the mean ± SEM of three assays. **(E, F)** Representative dot plots of expression of PD-L1 in MC-C **(E)** and LMM3 **(F)** tumor and tumor-infiltrating cells. **(G)** Acquired capacity of LMM3 lysate to promote the maturation of dendritic cells (DC) by pre-treatment with JSI-124. Expression of cell-surface receptor CD86 was evaluated by flow cytometry. DC were incubated with LMM3 lysate or with lysate from LMM3 cells that had been pre-treated in vitro for 24 h with different concentrations (1, 5, 10, and 20 ng/ml) of JSI-124. DC incubated with LPS served as a positive control. Negative controls were immature DC (DCi). Data represent the mean ± SEM of three independent experiments. **(H)** Vaccinating capacity against LMM3 of DC stimulated with a lysate from LMM3 cells that had been treated in vitro with 20 ng/ml of JSI-124. Two doses of DC of the different groups were inoculated in the footpad of mice 14 and 7 days before the s.c. challenge with different doses of LMM3 tumor cells. Vaccinating capacity was measured as an increase of tumor dose 50 (DT_50_) of LMM3 tumor in treated mice compared to control. Data represent the mean ± SEM of two independent experiments. In each experiment, 20–25 mice per group were utilized. Statistical comparison between experimental groups and DCi: ## *p* < 0.01; ### *p* < 0.001. Statistical comparison among experimental groups: ** *p* < 0.01; *** *p* < 0.001

MC-C tumor immunogenicity was associated with the capacity of MC-C tumor lysate to promote the maturation of DC as evaluated by the up-regulation of cell surface receptors CD80, CD86 and MHC II as well as production of the inflammatory cytokines TNF-α and IL-12p70 (Fig. 1B, C and D). In addition, this capacity of MC-C tumor lysate was correlated to its high concentration of HGMB1 and Hsp60 – two recognized danger signals that favor DC maturation – and low concentration of IL-10 and TGF-β – two known immunosuppressive cytokines that inhibit DC maturation [1, 24]; (Fig. 1E and F). In turn, MC-C tumor exhibited low constitutive expression of phosphorylated STAT3 (pSTAT3) – an activated molecule that is involved in the transcription of genes that induce tolerogenic and immunosuppressive signals [16]; (Fig. 2A and B ) – and low expression of surface PD-L1 in tumor cells and tumor infiltrating cells (Fig. 2C, D, and E).

On the other hand, LMM3 tumor displayed weak (if any) immunogenicity (Fig. 1A), which was associated with the incapacity of LMM3 tumor lysate to promote maturation of DC (Fig. 1B, C and D). This incapacity was correlated to a low concentration of HGMB1 and Hsp60 and a high concentration of IL-10 and TGF-β present in LMM3-tumor lysate (Fig. 1E and F). LMM3 tumor exhibited high constitutive expression of pSTAT3 (Fig. 2A and B ) and high expression of surface PD-L1 in tumor cells and tumor infiltrating cells (Fig. 2C, D and F).

The immunosuppressive properties of LMM3 tumor lysate were confirmed by its ability to counteract the capacity of LPS to promote DC maturation. In contrast, lysates prepared from normal spleen cells or MC-C tumor cells did not counteract LPS capacity (*Suppl. Figure *2).

Tumor lysate prepared from LMM3 tumor cells pre-treated with a natural inhibitor of pSTAT3 called JSI-124 acquired a significant capacity to promote the maturation of DC in a dose-dependent manner (Fig. 2G). In turn, pre-treatment of mice with these DC produced a significant protective effect (preventive vaccination) against the growth of live tumor cells implanted thereafter (Fig. 2H). A vaccinating similar effect was achieved with lethally-irradiated LMM3 tumor cells that had been pre-treated – before being irradiated—either with JQ1 (to inhibit the expression of PD-L1 or JSI-124 (to inactivate pSTAT3). These protective effects were tumor-specific and T-cell dependent (not shown).

The above considerations suggest that both chemically-induced MC-C and spontaneous LMM3 tumors bear specific antigens. However, in LMM3, these antigens seemed to be hidden by immunosuppressive signals released by the own tumor cells. It is worth noting that, even counteracting the mechanisms that prevent the onset of an anti-LMM3 tumor immune response, the magnitude of both the maturation of DC by LMM3 lysate and the preventive vaccinations was always several orders lower than that achieved with MC-C tumor. It suggests that the strength of MC-C tumor antigens is much greater than that of LMM3 ones.

### Contrasting effects of immunotherapies on growing tumors

When MC-C tumor cells were inoculated in naïve mice, tumor-bearing mice produced a significant anti-tumor immune response (although not strong enough to inhibit the growing tumor) characterized by classical markers of anti-tumor immunity (*Suppl. Figure *3). This significant immune response was, in turn, correlated to *a)* a relatively low expression of PD-L1 on the surface of tumor-infiltrating CD11b^+^ myeloid cells (Fig. 2E) and *b)* low expression of PD-1 in the surface of both T CD8^+^ and CD4^+^ splenic lymphocytes (*Suppl. Figure *4).

On the other hand, when LMM3 tumor cells were inoculated in naïve mice, tumor-bearing mice produced a weak (if any) anti-tumor immune response (*Suppl. Figure *3), which was correlated to *a)* high expression of PD-L1 on the surface of tumor-infiltrating CD11b^+^ myeloid cells (Fig. 2F) and *b)* increased expression of PD-1 in the surface of both T CD8^+^ and CD4^+^ splenic lymphocytes (*Suppl. Figure *4).

Based on these observations and on the fact that LMM3 tumor cells and tumor infiltrating cells displayed a significantly higher expression of PD-L1 than MC-C tumor cells (see above Fig. 2C, D, E and F), it was expected that the blockade of the PD-1/PD-L1 pathway might be more useful to treat LMM3 than MC-C growing tumors by unlocking the onset of an otherwise almost inexistent anti-tumor immune response. On the other hand, the blockade of CTLA-4 might be more beneficial to treat MC-C than LMM3 growing tumors by enhancing an ongoing anti-tumor immune response.

Our experiments confirmed these expectations. However, in both models, the combined treatment with anti-CTLA-4 and anti-PD-L1 was better than each separately.

**Fig. 3:**
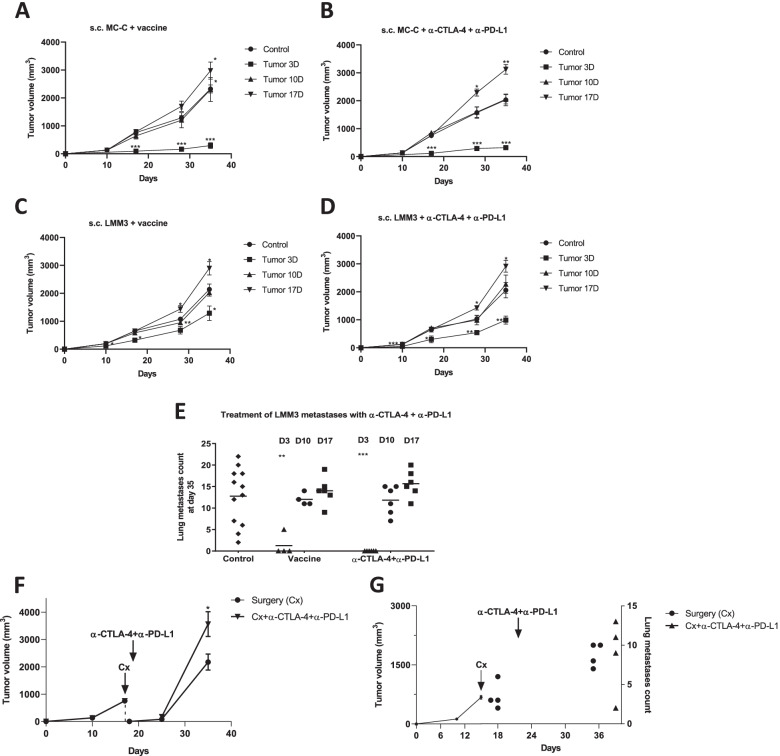
Therapeutic antitumor immunological schedules against growing and residual tumors.** (A, C)** Antitumor vaccines. 1 × 10^5^ MC-C **(A)** or LMM3 **(C)** cells were inoculated s.c. in the right flank. Dendritic cells (DC) were incubated with LMM3 lysate or with lysate from LMM3 cells that had been pre-treated in vitro for 24 h with different concentrations (1, 5, 10, and 20 ng/ml) of JSI-124. DC incubated with LPS served as a positive control. Negative controls were immature DC (DCi). Mice received in the left flank an antitumor vaccine (DC stimulated in vitro with MC-C lysate **(A)** or DC stimulated in vitro with LMM3 lysate from cells that had been treated in vitro with 20 ng/ml of JSI-124 **(C)**, starting at day 3, 10 or 17 of tumor growth (Tumor 3D, 10D and 17D, respectively). Control mice were inoculated with immature DC (DCi). **(B, D).** Immune checkpoint inhibitors (ICI). 1 × 10^5^ MC-C **(B)** or LMM3 **(D)** cells were inoculated s.c. in the right flank. Afterward, mice received anti-CTLA-4 + anti-PD-L1, starting at day 3, 10, or 17 of tumor growth. For simplicity, groups treated with anti-CTLA-4 alone and anti-PD-L1 alone were omitted. **(E)** Effect of vaccines and ICI on the growth of lung metastases of LMM3. Mice were treated with anti-CTLA-4 + anti-PD-L1, starting at day 3, 10, or 17 of tumor growth. Groups of mice that did not receive any treatment served as controls. **(F, G)** Therapeutic antitumor immunological schedules in MC-C local recurrences **(F)** and postsurgical LMM3 lung metastases **(G)**. Different groups of mice received anti-CTLA-4 + anti-PD-L1, starting the day after surgery. Each dose of anti-CTLA-4 and anti-PD-L1 was 100 μg. Anti-CTLA-4 was inoculated three times a week and anti-PD-L1 for 9 consecutive days, both i.p. Cx = surgery. Data from Figures A, B, C, D, F, and G represent the mean ± SEM of two or three independent experiments. 4—6 mice per group were utilized. Statistical comparison between experimental groups and control: * *p* < 0.05 **; *p* < 0.01; ***; *p* < 0.001

In the MC-C tumor model, vaccines based on lethally-irradiated MC-C tumor cells or DC stimulated by MC-C tumor lysate produced inhibitory effects on growing MC-C tumors somewhat similar to those achieved with ICI (Fig. 3 A and B).

On the other hand, no impact on growing LMM3 tumors was obtained with lethally-irradiated LMM3 tumor cells or DC stimulated with LMM3 lysate. Only the use of DC stimulated with LMM3 lysate prepared with inactivated STAT3 produced some anti-tumor effect although lower than that attained with ICI in both subcutaneous tumors and lung metastases (Fig. 3C, D and E).

In summary, as shown comparatively in Figs. 3A - D, the immunologic-mediated antitumor effects were stronger on the strongly immunogenic tumor (MC-C) than on the weakly immunogenic one (LMM3).

It is worth noting that the striking success of all of these immunologic treatments was achieved against incipient (≤ 10 mm^3^) but not larger growing tumors. In fact, in mice bearing large-sized tumors (mean volume: 650—800 mm^3^) – either strongly or weakly immunogenic – these treatments not only did not produce any inhibitory effect but an enhanced tumor growth (Fig. 3A - D) while no effect was observed on metastases (Fig. 3E). Attempting to get an effective treatment against these tumors, we doubled the doses of ICI. However, the results were worse than before since, upon this double dose treatment, all mice showed severe manifestations of auto-immunity and reached the experimental endpoint rapidly after the last dose (not shown).

### Contrasting outcomes of immunological strategies on incipient and residual tumors

Although inefficient to inhibit mid- and large-sized tumors, the ability of ICI to restrain the growth of incipient tumors might still have great clinical potential value if it were demonstrated that residual tumors- supposedly the targets of ICI in clinical settings – would behave in the same way as incipient tumors regarding their sensitivity to immunologic treatments. To test this contention, we used two clinically relevant tumor models:

- A model of local recurrence after subcutaneous (s.c.) MC-C tumors (650–800 mm^3^) were surgically excised, leaving intact the underlying skin. In these conditions, recurrent tumors become apparent one week after surgery in 100% of cases.
- A model of lung metastases after s.c. LMM3 tumors (650–800 mm^3^) were radically removed together with the underlying skin when spontaneous metastases were already established in the lung. Local tumors do not re-grow in these conditions, but all mice die with multiple metastases within a month after surgery.

As shown in Fig. 3 F and G, the growth of local recurrences and metastases was not inhibited by the very same treatments that strongly inhibited the growth of incipient tumors. Actually, the development of local tumor recurrences upon treatment with ICI was enhanced in the same way as large-sized tumors – from which residual tumors were derived by surgical debulking (Fig. 3F) – while the growth of metastases was similar in both treated and control groups (Fig. 3G).

### Simultaneous enhancement of large-sized tumors and inhibition of secondary tumor implants upon treatment with immune-checkpoint inhibitors

Enhancement of large-sized and residual tumors upon treatment with ICI and vaccines could be explained, at first sight, by an immunotherapy-mediated down-regulation of the antitumor immune response produced at the local tumor area [9, 32]. If this were the case, the growth rate of such enhanced tumors could get close to that attained in constitutive immune-deficient nude and NSG mice, but actually, it was significantly higher than in the latter (*Suppl. Figure 5*). These results suggested that other explanations were necessary. We evaluated the immunologic state of mice bearing large-sized tumors after treatment with ICI to account for this fact. Tumor-bearing mice produced an anti-tumor immune response (*Suppl. Figure *3) but only up to tumor volume reached 500 mm^3^. Afterwards, such immune response – either significant (for MC-C) or weak (for LMM3) – was sharply down-regulated.

**Fig. 4:**
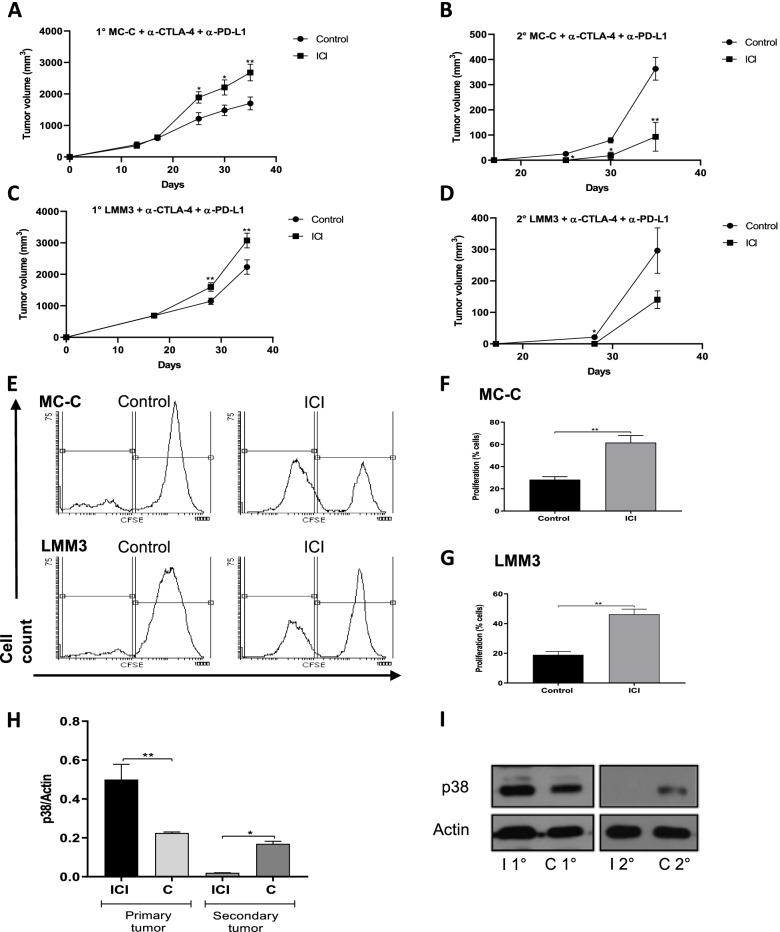
Simultaneous effects of immune checkpoint inhibitors (ICI) on primary large-sized tumors and secondary tumor-bearing mice.** (A, B, C, D)** Mice bearing MC-C **(A)** or LMM3 tumor **(C)** measuring 800 mm^3^ (1°MC-C or 1°LMM3, respectively) were challenged at day 17 with a secondary tumor implant **(B, D)** carried out in the contralateral flank (2°MC-C or 2°LMM3, respectively) and, simultaneously, treated with ICI (anti-CTLA-4 + anti-PD-L1). 5 × 10^5^ tumor cells were inoculated for MC-C and LMM3 primary tumors, and 2 × 10^5^ tumor cells for secondary implants. For each tumor, the figure shows a representative experiment (*n* = 4–6 mice per group) out of two experiments that rendered similar results. Data were expressed as mean (mm^3^) ± SEM of tumor volume. **(E)** Representative CFSE flow cytometric histograms of splenic T-cells from MC-C and LMM3 primary and secondary tumors bearing mice. **(F, G)** Percentage of the proliferation of splenic T cells from MC-C **(F)** and LMM3 **(G)** primary and secondary tumors bearing mice. Mice were treated with anti-CTLA-4 + anti-PD-L1 (immunized group), and non-treated mice served as control (control group). Each value represents the mean ± SEM of two assays. Each dose of anti-CTLA-4 and anti-PD-L1 was 100 μg per mouse. For simplicity, groups treated with anti-CTLA-4 alone and anti-PD-L1 alone were omitted. **(H, I)** Expression of phosphorylated (p)-38 (p38) by Western blotting. Macrophages (3 × 10^6^ cells) were collected surrounding the s.c. primary and secondary MC-C tumors 7 days post-secondary implant. Mice were treated with anti-CTLA-4 + anti-PD-L1 (immunized group), and non-treated mice served as control. A representative experiment is shown. The figure shows levels of p38 in the different groups, normalized with beta-actin densitometric units, representing the mean ± SE of three independent experiments. Statistical comparison between experimental groups and control: * *p* < 0.02; ** *p* < 0.01; *** *p* < 0.001. Immunized primary tumor (I 1°), immunized secondary tumor (I 2°), control primary tumor (C 2°) and control secondary tumor (C 2°).

A simple and reliable in vivo marker of anti-tumor immunity is the “concomitant immunity” phenomenon by which tumor-bearing mice are resistant to secondary tumor implants by a specific T-cell dependent mechanism (see *Suppl. Figure 3)*. As shown in Fig. 4 A-D, upon treatment with anti-CTLA-4 and anti-PD-L1, “concomitant immunity” was partially recovered in large-sized tumor-bearing mice. Another marker of anti-tumor immunity, such as tumor-antigen specific splenic T-cell proliferation, was also partially recovered upon treatment (Fig. 4E - G). In same way, after ICI therapy, percentage of splenic CD4^+^ and CD8^+^ T cells increased 16% and 32%, respectively, and, reciprocally, CD25^+^/FOXP3^+^ T regs dropped 31%.

Consequently, the enhancement of large-sized tumors upon treatment with ICI was, as paradoxical as it could be, associated with an increased anti-tumor immune response.

The stimulation of tumor growth by a relatively weak immune response has been claimed to be associated with the activation of TLR-4 and p38 pathway in macrophages attracted to the tumor place [16].

Confirming that claim, macrophages collected surrounding large-sized tumors from mice treated with ICI exhibited a significantly higher expression of p38 than that found in similar-sized tumors from non-treated mice or surrounding secondary tumor implants (Fig. 4H and I).

### Meta-tyrosine and blockade of p38 pathway enable an efficient anti-tumor therapy with anti-tumor vaccines and checkpoint inhibitors

Acceleration of tumor growth upon vaccines and ICI treatments might be counteracted by using two different but complementary strategies: a) SB 202190, a selective inhibitor of p38, to counteract the phenomenon of tumor-immune-stimulation; b) meta-tyrosine (m-Tyr), which, according to previously reported results [18] might restrain putative immune-checkpoints not counteracted by classical ICI and, in consequence, to act as an adjuvant for such immunologic therapies. The recovery effect of m-Tyr on splenic T-cell proliferation in immunosuppressed mice is shown in *Suppl. Figure 6.*

**Fig. 5:**
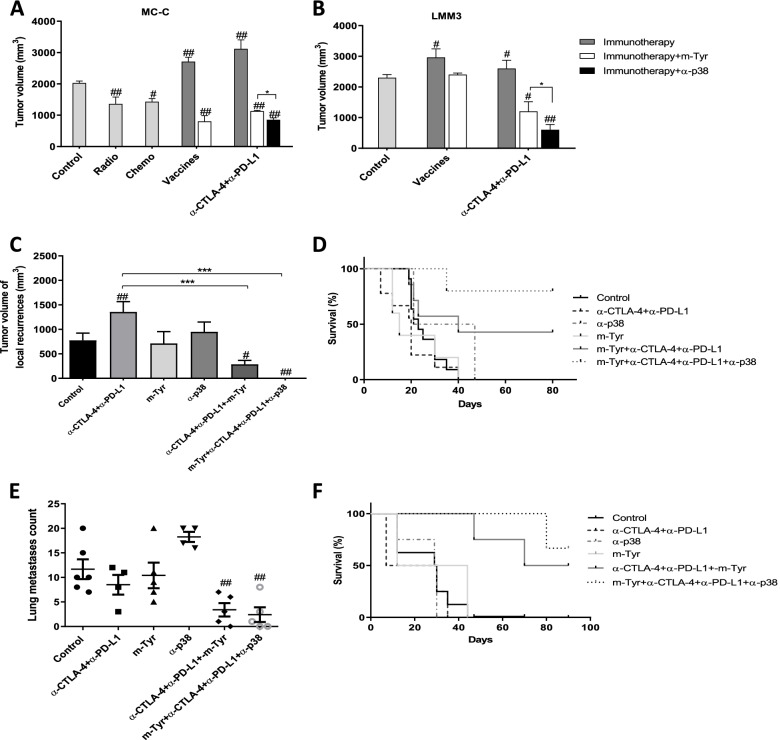
Effect of combined immunological treatment on growing and residual tumors.** (A, B)** 1 × 10^5^ MC-C **(A)** or LMM3 **(B)** cells were inoculated s.c. in the right flank. On day 17 of tumor growth, mice received immunotherapy [anti-CTLA-4 + anti-PD-L1, or antitumor vaccines (same schedules to Fig. 3)], immunotherapy plus m-Tyr (1.5 mg i.p. one dose, day 18) or immunotherapy plus anti-p38 (SB202190: 0.5 mg/kg i.p. for four days starting at day 18 of tumor growth). For MC-C, mice received a dose of radiotherapy (2000 grades) or vincristine (1 mg/kg i.p.). Tumor-bearing mice without treatment served as control. Tumor volumes were measured at day 35. **(C, D, E, F).** Effect of combined immunotherapy on local recurrences and metastases. Model of MC-C local recurrences: s.c. MC-C tumors (about 800 mm^3^) were surgically excised, leaving underlying skin (**C, D**) Model of lung metastases: s.c. LMM3 tumors (about 800 mm^3^) were radically removed with underlying skin when spontaneous metastases are already established **(E, F)** Mice were treated with immunotherapy (anti-CTLA-4 + anti-PD-L1, immunotherapy plus m-Tyr, immunotherapy plus SB202190 (on suture line for MC-C or i.v for LMM3) or immunotherapy plus both m-Tyr and SB202190. Operated mice without treatment served as control. Tumor volumes were evaluated two weeks after surgery. Survival percent were assessed 90 days after surgery. Data represent ± SEM of tumor volume (mm^3^) of 8—12 mice per group. For local recurrences and residual metastases, a sub-group was sacrificed 15 days after surgery to histological analysis and to count number of metastases. In all cases, the effects of treatments with m-Tyr or SB202190 alone were similar to control group and omitted for simplicity. Statistical comparison between experimental groups and control: # *p* < 0.05; ##: *p* < 0.01. Statistical comparison among experimental groups: * *p* < 0.05; ***; *p* < 0.001

As shown in Fig. 5A and B, the combined treatment not only counteracted the tumor enhancement effect by ICI but also achieved significant inhibition of large-sized tumors in both strongly and weakly immunogenic models. Antitumor vaccines produced similar effects to ICI, especially in the strongly immunogenic model. Furthermore, the combined treatment of ICI or vaccines with meta-tyrosine, and SB 202190 was even more effective than classical chemotherapy and radiotherapy against growing murine tumors (Fig. 5A). However, despite these promissory results, the tumors continued to grow – although significantly more slowly than controls – and all mice finally reached the experimental endpoint.

**Fig. 6:**
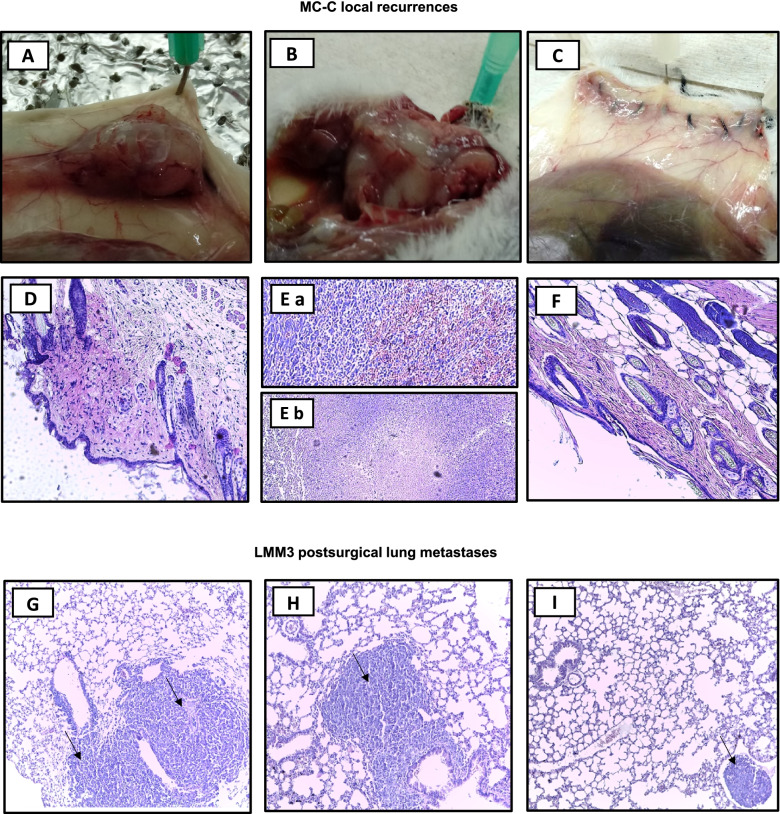
Representative macroscopic and histopathological images of tumor recurrences.** (A—F)** MC-C tumor local recurrences images (H&E staining, 100X) at day 15 post-surgical removal, corresponding to untreated control **(A, D)**, anti-CTLA-4 + anti-PD-L1 **(B, E)** and anti-CTLA-4 + anti-PD-L1 + m-Tyr + anti-p38 **(C, F)**. Noted the medium-size tumor of untreated mouse **(A)**, the large size tumor of a mouse treated with anti-CTLA-4 + anti-PD-L1 **(B)**, and an imperceptible tumor mass in the suture line from a mouse treated with anti-CTLA-4 + anti-PD-L1 + m-Tyr + anti-p38 **(C)**. **(G—I)** LMM3 post-surgical lung metastases images (H&E staining, 100X) at day 15 after surgery, corresponding to control **(G)**, anti-CTLA-4 + anti-PD-L1 **(H)** and anti-CTLA-4 + anti-PD-L1 + m-Tyr + anti-p38 **(I)** treated mice. Arrows point sites of tumor cells in the same field

A more striking effect was attained against the growth of local recurrences and metastases after surgical debulking the primary tumor. In effect, while local recurrences and residual metastases caused the death of 100% of non-treated controls or mice treated with each immunologic strategy separately, the combined treatment not only produced tumor-inhibitory effects but even it managed to cure about 80% of mice bearing local recurrences and about 60% of mice bearing residual metastases in the lung (Fig. 5C - F and Fig. 6). It is worth noting that, in all the schedules used herein, neither m-Tyr nor SB 202190 produced, on their own, any inhibitory effect but collaborate to make powerfully efficient an otherwise inefficient therapy with vaccines and ICI.

## Discussion

In the last 50–60 years, surgery, radio, and chemotherapy have improved the management of human cancer. However, the progress has been much slower than initially expected, mainly associated with the difficulty of treating local recurrent and disseminated cancer [33–35].

In this context, immunologic strategies mainly based on the blockade of immune-checkpoints, emerged as a real possibility to treat advanced cancer because they could theoretically overcome the limitations of conventional non-tumor specific anti-cancer therapies.

However, although immune-checkpoint inhibitors (ICI) improved the results achieved with conventional therapies on some clinical tumors, up to date, the overall benefits are supported by a relatively small percentage of patients. Contrastingly, many of them exhibit no significant improvement or even a condition called “hyperprogressive cancer disease” (HPD) with rapid tumor growth, increased metastatic load, and shorter survival times [10, 32, 36].

Based on these clinical results, it seems to be necessary not only to develop predictors of response to immunotherapy and rational combination therapies that can enhance their efficacy but also to elucidate the mechanisms underlying the phenomenon of HPD.

To this aim, in this work, we have assayed the therapy with anti-CTLA-4 and anti-PD-L1, as well as with classical antitumor vaccines, on two growing murine tumors with widely different degrees of immunogenicity, a strongly immunogenic chemically-induced fibrosarcoma and a weakly immunogenic and highly metastatic mammary adenocarcinoma of spontaneous origin. Treatment was initiated at various stages of tumor growth and assayed on the re-growth of residual tumors (local recurrences and metastases) after surgical tumor extirpation to mimic real clinic situations.

In tumor-bearing mice, the immune response was increased upon treatment with anti-tumor vaccines and, more efficiently, with a combination of anti-CTLA-4 plus anti-PD-L1 antibodies. This was reflected in an inhibition of both growing tumors proportionally to tumor immunogenicity.

However, these treatments were genuinely efficient only when they faced incipient tumors (< 10 mm^3^). Afterwards, no anti-tumor effects were attained. In fact, when treatment started at the time when tumor was large (> 500 mm^3^), enhancement of tumor growth was achieved in both tumor models. In addition, when treatments were assayed on residual local tumors after surgical large tumor excision, the growth of residual tumors was enhanced in the same way as large-sized tumors from which they were derived. Regarding metastases, neither inhibitory nor stimulatory effects were observed upon treatment. These results indicated that a residual tumor, even composed of a similar number of cells to that of an incipient tumor, behaves, concerning its sensitivity to immunologic strategies, much more like a large than an incipient tumor. As a corollary, data suggest that incipient tumors are not good models to predict the outcome of immunological therapies on residual tumors.

The lack of therapeutic response or even an accelerated growth of non-incipient murine tumors after these immunologic treatments may be paralleled with the lack of response, or the HPD observed in some patients with advanced cancer who have received a therapy with ICI. The mechanisms underlying these undesired therapeutic responses remain speculative. Recent work supports the idea that HPD after therapy with ICI may be more frequent in patients with MDM2 family amplification and EGFR aberrations than patients without them [37]. Another work identified increased expression of oncogenic pathways and mutations in known tumor suppressor genes such as VHL and TSC2 in tumor cells displaying HPD after therapy with anti-PD-1 therapy [38]. Others have proposed that, even though treatment with anti-PD-1/PD-L1 and anti-CTLA-4 antibodies would usually expand anti-tumor CD8 + and CD4 + T cells, such treatment might, upon certain circumstances, increase the population of PD-1 + T regs producing an effect of immunosuppression. Kamada et al. [39] found that in non-HPD patients, the ratio of T-regs/CD8 + cells, the proportion of Ki67 + T-regs/ Ki67 + CD8 + cells and the percentage of Ki67 + T-regs decreased significantly after nivolumab treatment. At the same time, they remained stable or even reduced in HPD patients. In fact, PD-1 + Tregs and especially M2-like macrophage infiltration induced by anti-PD-1 antibodies have been recently considered a major hallmark of HPD in clinical settings [10, 11]. In the same line, a correlation between decreased immunogenicity and HPD has been proposed (40).

Although all of the predictors and mechanisms suggested above may play a role in some cases, it is difficult to attribute to them a general role. In effect, in our experiments, differences associated with different genetic backgrounds in the tumor-bearing host are unlikely since all tumor-bearing hosts were inbred mice. In addition, the tumors that displayed hyperprogressive growth upon therapy with ICI behaved like “normal” tumors (that is, not HPD tumors) when they were transplanted into naïve mice, suggesting that no mutations occurred before or during the phase of accelerated tumor growth. In the same way, if a state of immunosuppression were the explanation for the tumor-accelerating effect produced by immunologic strategies on large or local recurrent tumors, the growth of such tumors could get relatively close to that attained in immune-depressed nude and extremely immune-deficient NSG mice but not to grow faster than the latter as it actually occurred. Further, the enhancement of large-sized and residual tumors upon immunological treatments was achieved, surprisingly, in the face of an increased anti-tumor immune response. Lastly, although low immunogenicity may favor HPD in some cases, in this work, accelerated tumor growth after immunologic treatment was observed associated with both strongly and weakly immunogenic tumors.

A putative explanation for the acceleration of tumor growth upon current immunological therapies might be attained on the basis of the theory of tumor-immunostimulation, stated by Prehn many years ago [19]. That theory postulates that the antitumor immune response would not be linear, as the orthodoxy predicts, but biphasic with “strong” immune responses producing inhibition, “weak” responses inducing acceleration of tumor growth and “very weak” ones producing no effect (see the *Suppl. Figure 1* for a better understanding of the phenomenon). This proposal suggests that immunotherapy against cancer may produce, in the highly immunosuppressive microenvironment of large tumors, weak immune responses that would promote rather than inhibit tumor growth [16, 19, 27, 41]. In effect, when tumors have surpassed the critical volume of 500 mm^3^, tumor-bearing mice usually enter into a state of systemic immune-depression against tumor antigens historically known as “immunological eclipse” that, according to our observations concerning the kinetics of the primary tumor and secondary tumor implants, would be more robust near the primary tumor than anywhere else on the body. That state of immunosuppression is presumably not reversed by incomplete surgical resection because, as we pointed above, recurrences behave, as for their sensitivity to immunologic treatments, much like the large tumors from which they were derived. At that tumor stage, the magnitude of the antitumor immune response near the tumor site could be considered, before any immunologic treatment, as “very weak” and placed near “0” on the biphasic antitumor immune response curve (see *Suppl Fig. 1*). When an immunologic treatment is utilized against these large tumors (and also against local recurrences), it would produce a relatively weak increase of the immune reaction, moving it to the right on the curve, for example toward “c”, producing accelerated tumor growth. The observation that metastases from large tumor-bearing mice were neither inhibited nor stimulated upon the very same immunologic treatments that accelerated the primary tumor could be similarly explained by assuming that the state of immunosuppression is less profound far from the primary tumor, where metastases would be established. In consequence, in such places, the basal antitumor immune response would be, for example, near “a” and after the immunologic treatment it would be similarly increased as before, moving the immune response towards “e”, where neither inhibitory nor stimulatory effects are expected.

Although antitumor vaccines and ICI did not produce on their own inhibitory effects on large tumors or their metastases, more stringent strategies, for example by incorporating new and potent adjuvants to the treatment, could move the immune reaction beyond the stimulatory zone up to the inhibitory part of the curve (for example near “f”). In our experiments this role was achieved by meta-tyrosine (m-Tyr), an unnatural isomer of tyrosine. Former experiments had demonstrated that high concentrations of m-Tyr, chronically administered by the intravenous route, could directly inhibit tumor cell proliferation through inactivation of pSTAT3 and down-regulation of both the NFκB/NOTCH axis and survivin expression (22, 42). More recent experiments demonstrated that m-Tyr, when administered once or few times by the intraperitoneal route, as it was used herein, does not produce any direct antitumor effect but it may boost the overall immune response against different antigens and rescue the organism from states of immunosuppression not counteracted by anti-CTLA-4 and anti-PD-L1 antibodies [18]. On this basis, when we combined this schedule of m-Tyr with antitumor vaccines or ICI, a significant inhibitory effect on non-incipient tumors was observed.

Another strategy to overcome the limitations of current immunologic therapies could involve the counteraction of the proper phenomenon of tumor immunostimulation. This phenomenon has recently received a mechanistic interpretation [16] according to which a weak antitumor immune response would promote tumor growth upon enhanced activation of p38 signaling pathway in macrophages recruited at the tumor site. The fact that tumor infiltration by M2 macrophages is a common finding in clinical cancer displaying HPD after treatment with ICI [10] further supports the putative involvement of the phenomenon of tumor immunostimulation in those cases. On this basis, when we combined vaccines or ICI with a specific inhibitor of p38, a significant inhibitory effect on large tumors was observed. In former works [1, 16], immunotherapeutic strategies were reported to be improved by the use of non-specific anti-inflammatory agents such as indomethacin or low doses of dexamethasone. However, in our assays the anti-inflammatory agent SB202190, specific against p-38, rendered better results.

In our hands, the best therapeutic results were accomplished by combining ICI with both m-Tyr and SB202190 to treat local tumor recurrences and metastases after surgery. This combined therapy produced a profound inhibition of the tumor growth that resulted in 80% of cures of local recurrent tumors from the strongly immunogenic tumor, and in about 60% of cures of metastatic residual tumors from the weakly immunogenic one, in a context where, no treatment produced 100% of deaths in both cases and treatment with ICI alone produced not only 100% of deaths but also hyperprogressive or accelerated tumor growth in the case of local recurrent tumors. It is worth to note that the combined therapy utilized in this work was significantly better not only than current immunologic approaches but also than conventional chemotherapy and radiotherapy. The fact that neither m-Tyr nor the specific inhibitor of p38 pathway alone produced any antitumor effect suggested that they did not act on their own but they collaborate with the current immunologic therapies allowing an otherwise ineffective immunologic strategy to have a chance to be effective. Further characterization of the T cells activity upon the combined treatment is necessary to understand more accurately this promising anti-tumor effect. In clinical trials for advanced cancer, ICI and other immunologic approaches only evidenced significant beneficial effects in a limited cluster of patients [43–46]. We suggested that these patients might exhibit stronger immune reactions than the general population or, alternatively, they have been unable to mount a significant macrophage-related-pro-tumorigenic TLR-4 and p-38 dependent inflammatory response preventing the emergence of a state of immunostimulation. The observation presented in a former paper [16] that the immunostimulatory arm of the immune response curve was not observed in Winn assays carried out in macrophage-depleted and TLR-4 knock-out mice, seems to support this suggestion. In fact, the therapeutic antitumor success (when it occurred) of BET (bromo-domain and extra-terminal motif) inhibitors could be associated, at least in part, with their ability to impair macrophage-mediated inflammation [44].

In summary, there is great interest in developing methods and markers that can identify patients and tumor types that could get benefit from different schedules of immunotherapy. In fact, hundreds of trials have been initiated in the last few years and many of them are still ongoing including ICI or new cancer vaccines either working alone or combined with chemotherapy, radiation therapy, targeted therapy, intra-tumoral therapy, novel immunomodulators, bispecific and multispecific antibodies, microbioma modulators, adoptive cell therapy including chimeric antigen T-cell receptors and other novel strategies [45, 46]. In this context, the analysis of genetic profile of tumor antigens, the search for new adjuvants that can blockade new checkpoints not counteracted by already known ICI (m-Tyr is an example of them) and a deeper understanding of the phenomenon of tumor immunostimulation could also contribute to improve the current therapies against cancer.

## Supplementary Information

**Additional file 1. **

**Additional file 2: Supplementary Figure 1**. Idealized monotonic (**A**) and biphasic (B) anti-tumor immune reaction curve. **Supplementary Figure 2**. Counteracting effect of LMM3 lysates on the capacity of LPS to promote the maturation of DC. **Supplementary Figure 3**. Classical markers of antitumor immunity in MC-C and LMM3 tumor-bearing mice. **Supplementary Figure 4**. Expression of PD-1 in CD4^+^ (**A**, **B**, **C**) and CD8^+^ (**D**, **E**, **F**) splenic lymphocytes of MC-C and LMM3 bearing mice. **Supplementary Figure 5**. (**A**, **C**)Kinetics of MC-C and LMM3 growing tumors in euthymic, nude, and NSG mice. **Supplementary Figure 6**. Percentage of *in vitro *proliferation of splenic CD3^+^ cells.

## Data Availability

All data generated or analysed during this study are included in this published article and its supplementary information files.

## Abbreviations

CFSE: Carboxi fluorescein diacetate succinimidyl ester
CICUAL: Committee for the care and use of laboratory animals
CTLA-4: Cytotoxic T-lymphocyte-associated protein
DC: Dendritic cells
HPD: Hyperprogressive-cancer disease
ICI: Immune-checkpoint inhibitors
IR: Immune response
M-Tyr: Meta-tyrosine
NSG: NOD scid gamma
PD-1: Programmed cell death-1
PD-L1: PD ligand-1
TD50: Tumor dose 50
TGR: Tumor growth rate
