# Supplementary material for: Inhibition of hyperprogressive cancer disease induced by immune-checkpoint blockade upon co-treatment with meta-tyrosine and p38 pathway inhibitor
Source: BMC Cancer. 2022 Aug 3;22:845. doi: 10.1186/s12885-022-09941-2 (PMC9347122; doi:10.1186/s12885-022-09941-2)
Supplement: Supplementary file 1 — Additional file 1. [file 12885_2022_9941_MOESM1_ESM.pdf]

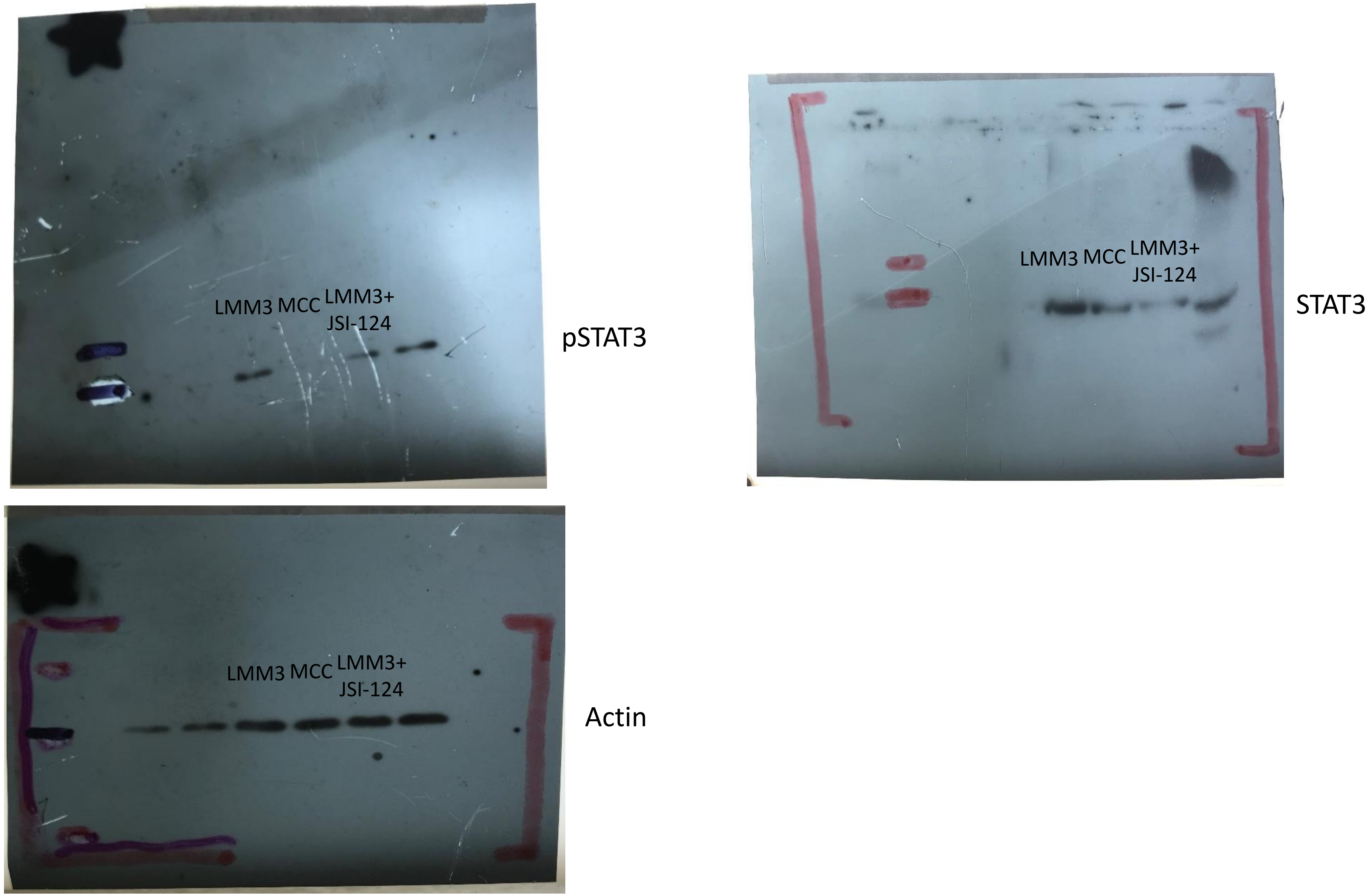

Figure 2: Expression of activated STAT3 (pSTAT3) in MC-C and LMM3 tumors. pSTAT3 was determined by Western Blotting in MC-C, LMM3 tumor cells and LMM3 cells that have been treated with 20 ng/ml of JSI-124 Controls with actin and total STAT3 were added. Results are representative of three similar experiments.

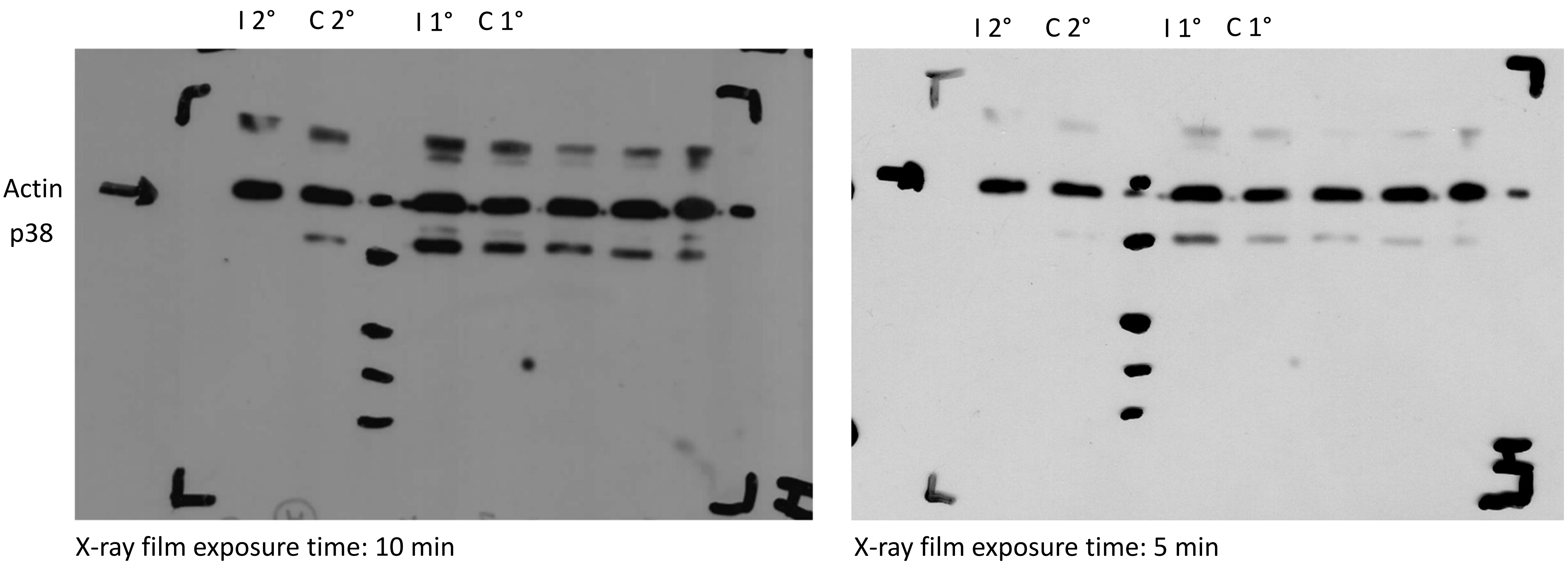

Figure 4: Expression of phosphorylated (p)-38 (p38) by Western blotting. Macrophages (3×10^6 cells) were collected surrounding the s.c. primary and secondary MC-C tumors 7 days post-secondary implant. Mice were treated with anti-CTLA-4 + anti-PD-L1 (immunized group), and non-treated mice served as control.

Immunized primary tumor (I 1°), immunized secondary tumor (I 2°), control primary tumor (C 1°) and control secondary tumor (C 2°) groups.
