# Supplementary material for: Inhibition of hyperprogressive cancer disease induced by immune-checkpoint blockade upon co-treatment with meta-tyrosine and p38 pathway inhibitor
Source: BMC Cancer. 2022 Aug 3;22:845. doi: 10.1186/s12885-022-09941-2 (PMC9347122; doi:10.1186/s12885-022-09941-2)
Supplement: Supplementary file 2 — Additional file 2: Supplementary Figure 1. Idealized monotonic (A) and biphasic (B) anti-tumor immune reaction curve. Supplementary Figure 2. Counteracting effect of LMM3 lysates on the capacity of LPS to promote the maturation of DC. Supplementary Figure 3. Classical markers of antitumor immunity in MC-C and LMM3 tumor-bearing mice. Supplementary Figure 4. Expression of PD-1 in CD4+ (A, B, C) and CD8+ (D, E, F) splenic lymphocytes of MC-C and LMM3 bearing mice. Supplementary Figure 5. (A, C)Kinetics of MC-C and LMM3 growing tumors in euthymic, nude, and NSG mice. Supplementary Figure 6. Percentage of in vitro proliferation of splenic CD3+ cells. [file 12885_2022_9941_MOESM2_ESM.docx]

**SUPPLEMENTARY FIGURES**

**Supplementary Figure 1: Idealized monotonic (A) and biphasic (B) anti-tumor immune reaction curve.** Graphics relate the quantity of anti-tumor immune cells to the quantity of target tumor cells [x-axis] with tumor growth [y-axis]. Note that *a, b, c, d, e, f,* and *g* are letters that indicate different ratios between the number of immune reactants and number of target tumor cells, with *a* and *g* representing, respectively, the lowest and the highest ratios tested and with *b, c, d, e* and *f* representing intermediate values between *a* and *g*.

**Supplementary Figure 2**: **Counteracting effect of LMM3 lysates on the capacity of LPS to promote the maturation of DC.** DC were incubated with LMM3 lysate alone and with a mixture of LPS (5 µg/ml) and LMM3 lysate. Controls were immature DC (DCi). DC incubated with a mixture of LPS and lysate from normal spleen cells, or MC-C tumor exhibited similar values to that obtained with DC+LPS. For simplicity, these data were omitted from the figure. Data represent the mean ± SEM of three independent experiments. Statistical comparison between: Experimental groups vs. Control (DCi):###: p < 0.001. Statistical comparison among experimental groups: *: p < 0.05; ***: p < 0.001.

**Supplementary Figure 3: Classical markers of antitumor immunity in MC-C and LMM3 tumor-bearing mice.** **(A)** Concomitant immunity is expressed as the ratio between tumor dose 50 (TD_50_) of secondary tumor in tumor-bearing mice /TD_50_ in control mice. Tumor volume in the right flank when the secondary tumor was implanted was 400 mm^3^ in both MC-C and LMM3 tumor-bearing mice. The dashed line [value=1] represents TD_50_ in control mice. Each bar represents the mean ± SEM of n experiments. n=5 for experiments in euthymic mice. The primary tumor was MC-C, and the secondary tumor was MC-C, or the primary tumor was LMM3, and the secondary tumor was LMM3. n=2 for experiments in nude mice. The primary tumor was MC-C, and the secondary tumor was MC-C, or the primary tumor was LMM3, and the secondary tumor was LMM3. n=2 for experiments in euthymic mice. The primary tumor was MC-C, and the secondary tumor was LMM3, or the primary tumor was LMM3, and the secondary tumor was MC-C. **(B)** Winn test. Experimental group: 50x10^6^ spleen cells from mice bearing either MC-C or LMM3 tumors measuring 400 mm^3^ were mixed, in *vitro*, with 5x10^5^ MC-C or LMM3 tumor cells, respectively, (ratio 100:1) and the mixtures were s.c. inoculated in naive mice. Control group: 50x10^6^ spleen cells from normal mice were mixed, in *vitro*, with 5x10^5^ MC-C or LMM3 tumor cells, respectively, and the mixtures were s.c. inoculated in naive mice. Percentage of tumor growth inhibition in experimental groups compared with control groups at day 35 after tumor inoculation was expressed as % tumor inhibition: [(1 - tumor volume in experimental mice) / tumor volume in control mice] x 100.  Each bar represents the mean ± SEM of n experiments. n=5 for experiments in which spleen cells were obtained from MC-C or LMM3 tumor euthymic mice. n=2 for experiments in which spleen cells were obtained from MC-C or LMM3 tumor nude mice.  **(C)** Adoptive transference of immunity (only in euthymic mice). Normal mice received 1x10^8^ spleen cells by the i.p. route from mice bearing MC-C or LMM3 tumors measuring 400 mm3 (experimental group) or normal mice (control group). Two hours later, mice were challenged with 5x10^5^ MC-C or LMM3 tumor cells by the s.c. route. Each bar represents the mean ± SEM of six experiments for MC-C or two experiments for LMM3 tumor. Percentage of tumor growth inhibition in experimental groups compared with control at day 35 after tumor inoculation was expressed as % tumor inhibition: [(1 - tumor volume in experimental mice) / tumor volume in control mice] x 100. **(D)** Cell-mediated cytotoxicity assay (only in euthymic mice). The ability of spleen cells to specifically kill ^54^Cr-labeled tumor cells in *vitro.* Cytotoxic activity of 2x10^6^ spleen cells from normal (normal) or MC-C- or LMM3-bearing mice measuring 400 mm^3^ (experimental) against 2x10^4^ Cr-labeled MC-C or LMM3 tumor cells, respectively. Mean ± SEM of four experiments for MC-C or two experiments for LMM3 tumor. Specific lysis was calculated as: % lysis = [(experimental cpm - normal cpm) / (cpm with tryton - normal cpm)] x 100.

**Supplementary Figure 4**: **Expression of PD-1 in CD4^+^ (A, B, C) and CD8^+^ (D, E, F) splenic lymphocytes of MC-C and LMM3 bearing mice**. Results were expressed as a percentage of cells **(A, D)** and mean fluorescent intensity (MFI) **(B, E)**. Cell populations were evaluated by flow cytometry. **(C, F)** Representative flow cytometric histograms of the expression of PD-1. The Figure shows a representative experiment out of two experiments that rendered similar results. Data are expressed as mean ± SEM. Statistical comparison between: experimental groups vs. control: # p<0.05; ## p<0.01. Statistical comparison among experimental groups: *p<0.05; **p<0.01. n=5 mice per group.

**Supplementary Figure 5**: **(A, C) Kinetics of MC-C and LMM3 growing tumors in euthymic, nude, and NSG mice.** Tumor growth was initiated at day 0 with an s.c. inoculum of 1×10^5^ MC-C **(A)** or LMM3 **(B)** tumor cells in the right flank. On day 17 of tumor growth, tumor-bearing euthymic mice received immune checkpoint inhibitors anti-CTLA-4 (inoculated i.p. three times a week) + anti-PD-L1 (inoculated i.p. for 9 consecutive days) or an antitumor vaccine (4×10^6^ X-lethally irradiated MC-C tumor cells or LMM3 tumor cells that had been treated *in vitro* with 20 ng/ml of JSI-124). For simplicity, anti-CTLA-4 and anti-PD-L1 groups were excluded in each model. Tumor growth in nude and NSG that did not receive any treatment was also registered. Each dose of anti-CTLA-4 and anti-PD-L1 was 100 µg per mouse. **(B, D)** Tumor growth rate (TGR) = difference of tumor volumes/days. The TGR was calculated between the onset of treatment (day 17) and day 28. Data represent the mean of tumor volume ± SEM of two independent experiments. Statistical comparison between: Experimental groups vs. Control (Euthymic): ## p < 0.01; ### p < 0.001. Experimental groups vs. Control (Nude): * p < 0.05; *** p < 0.001.  Experimental groups vs. Control (NSG): γ p < 0.05.

**Supplementary Figure 6: Percentage of *in vitro* proliferation of splenic CD3^+^ cells.** Control mice (non-immunosuppressed), immunosuppressed mice (IS), and immunosuppressed mice treated with 1.5 mg of m-Tyr every 24 hours during 7 days (IS + m-Tyr) were evaluated. **(A)** Representative CFSE flow cytometric histograms of splenic T- cells, **(B)** Percentage of proliferation, and **(C)** percentage ofCD3^+^ splenic lymphocytes. Results are expressed as mean ± SEM. The experiment is representative of two independent experiments.
